# Effect of *Arthrospira maxima* Phycobiliproteins, Rosiglitazone, and 17β-Estradiol on Lipogenic and Inflammatory Gene Expression during 3T3-L1 Preadipocyte Cell Differentiation

**DOI:** 10.3390/ijms25147566

**Published:** 2024-07-10

**Authors:** Ruth Marina García-García, María Eugenia Jaramillo-Flores

**Affiliations:** Ingeniería Bioquímica, Escuela Nacional de Ciencias Biológicas, Instituto Politécnico Nacional, Mexico City CP 07738, Mexico; rgarciag1910@alumno.ipn.mx

**Keywords:** *Arthrospira maxima*, phycobiliproteins (PBPs), rosiglitazone (RSG), 17β-estradiol (E), inflammation, obesity

## Abstract

The study evaluated the effects of *Arthrospira maxima* phycobiliproteins (PBPs), rosiglitazone (RSG), and 17β-estradiol (E) on the differentiation process of 3T3-L1 cells and on their regulation of lipogenic and inflammatory gene expression at different stages of the process. The results showed that phycobiliproteins promoted cell proliferation after 24 h of treatment. Furthermore, for all three treatments, the regulation of the highest number of markers occurred on days 6 and 12 of differentiation, regardless of when the treatment was applied. Phycobiliproteins reduced lipid droplet accumulation on days 3, 6, 10, and 13 of the adipogenic process, while rosiglitazone showed no differences compared to the control. On day 6, both phycobiliproteins and rosiglitazone positively regulated *Acc1* mRNA. Meanwhile, all three treatments negatively regulated *Pparγ* and *C/ebpα*. Phycobiliproteins and estradiol also negatively regulated *Ucp1* and *Glut4* mRNAs. Rosiglitazone and estradiol, on the other hand, negatively regulated *Ppara* and *Il-6* mRNAs. By day 12, phycobiliproteins and rosiglitazone upregulated *Pparγ* mRNA and negatively regulated *Tnfα* and *Il-1β*. Additionally, phycobiliproteins and estradiol positively regulated *Il-6* and negatively regulated *Ppara*, *Ucp2*, *Acc1*, and *Glut4*. Rosiglitazone and estradiol upregulate *C/ebpα* and *Ucp1* mRNAs. The regulation exerted by phycobiliproteins on the mRNA expression of the studied markers was dependent on the phase of cell differentiation. The results of this study highlight that phycobiliproteins have an anti-adipogenic and anti-inflammatory effect by reducing the expression of adipogenic, lipogenic, and inflammatory genes in 3T3-L1 cells at different stages of the differentiation process.

## 1. Introduction

Adipocytes are the primary cells in adipose tissue and play a crucial role in maintaining energy balance. They are involved in processes such as lipogenesis and lipolysis, which allow for the storage and release of energy based on the body’s energy balance status. Additionally, these cells monitor lipid levels in the body and send signals that affect appetite, satiety, and sleep patterns [1]. Due to their function in energy metabolism, adipocytes are considered endocrine cells due to their ability to secrete adipokines, substances that have a significant impact on the immune system, blood vessels, and insulin sensitivity [2].

Excessive or inadequate growth of adipocytes is a risk factor for diseases such as obesity, cardiovascular diseases, diabetes, and cancer. Under these conditions, there is an increased amount of adipose tissue in the body and/or improper distribution. Therefore, maintaining a healthy balance in adipocyte size and distribution can help prevent these diseases [3]. Currently, there is great interest in the signals that affect the development and function of adipocytes. The regulation of the growth and differentiation of these cells requires a combination of factors, such as extracellular and environmental signals, intracellular and transcriptional effectors, and unknown serum factors. These combinatorial signals are essential for controlling the size and function of adipocytes [3]. The number of adipocytes present in an organism is largely determined by the process of differentiation of these cells [4], a process by which stem cells transform into specialized cells with a specific function. In the case of adipocytes, this process involves the formation of cells capable of storing fat and contributing to the increase in adipose tissue in the body [5].

Two of the compounds studied that have a direct effect on adipogenesis are rosiglitazone (RSG) and 17β-estradiol (E). RSG is one of the medications used for patients with type 2 diabetes (T2DM) to reduce insulin resistance and hyperglycemia by lowering blood glucose levels. Additionally, this drug induces adipose differentiation, triglyceride (TG) storage, and lipogenesis [6]. Furthermore, RSG induces white adipose tissue (WAT) transdifferentiation into brown adipose tissue (BAT) by increasing mitochondrial mass and lipid oxidation [7]. On the other hand, E has been recognized as an important factor in regulating adipose tissue metabolism in women [8]. Estradiol plays a significant role in the regulation of adipocyte differentiation and development. It has also been reported that E stimulates the proliferation of human preadipocytes, which can remain undifferentiated cells, into adipocytes [9].

Consuming bioactive compounds from the diet or dietary supplements is one way to control obesity [10], including cyanobacteria and algae, which are important resources for producing bioactive compounds such as pigments, proteins, and peptides with biological activity [11]. However, there are few studies on proteins or peptides regarding their suppression of the differentiation process from preadipocytes to adipocytes and whether they stimulate lipolysis and/or reduce the number of adipocytes through apoptosis [12]. Chlorophyll derivatives obtained from the cyanobacterium *Cyanobium* sp. [13], as well as a 60% ethanol extract of the marine algae *Grateloupia elliptica*, showed significant inhibition of lipid accumulation in 3T3-L1 cells [14].

In recent years, genus Arthrospira, formerly known as Spirulina, has gained attention as a potential source of valuable nutrients for the prevention and treatment of chronic diseases. Arthrospira is a cyanobacterium, and consists of two species, *A. platensis* and *A. maxima* [15]. These cyanobacteria belong to the phylum Cyanobacteria and are unicellular organisms previously known as “blue-green algae” [16]. The genus *Arthrospira* is rich in essential nutrients and contains pigment proteins such as phycobiliproteins (PBPs), which are a family of proteins that play an important role in light absorption in cyanobacteria, red algae, cryptomonads, and cyanelles [17]. Phycobiliproteins are water-soluble proteins with phycobilins (chromophores) covalently attached through cysteine residues [18]. Phycocyanin and allophycocyanin are PBPs that have been studied for their anticancer, antioxidant, anti-inflammatory, and hepatoprotective properties, among others. In addition to its nutritional value, Arthrospira has also been used in cosmetic applications and has been recognized by the United States Food and Drug Administration (FDA) as a safe ingredient [19].

Furthermore, a 70% ethanol extract of *A. maxima*, with chlorophyll a and c-phycocyanin as its main components, reduced lipid droplets and decreased adipogenic proteins such as C/EBP*α*, PPARγ, and aP2, as well as lipogenic proteins including SREBP1, ACC, FAS, LPAATβ, Lipin1, and DGAT1, after 8 days of treatment [20]. However, there are few reports that evaluate the effect of PBPs on the expression of a wide variety of genes involved in the inflammatory process in obesity and in the differentiation process of adipocytes. Based on the above, this study aimed to evaluate the effect of *A. maxima* phycobiliproteins, versus rosiglitazone and 17β-estradiol, on the differentiation process of 3T3-L1 cells and their regulation of lipogenic and inflammatory gene expression at different time points.

## 2. Results

### 2.1. Effect of A. maxima Phycobiliproteins (PBPs) on Cell Proliferation of 3T3-L1 Preadipocytes

**Figure 1:**
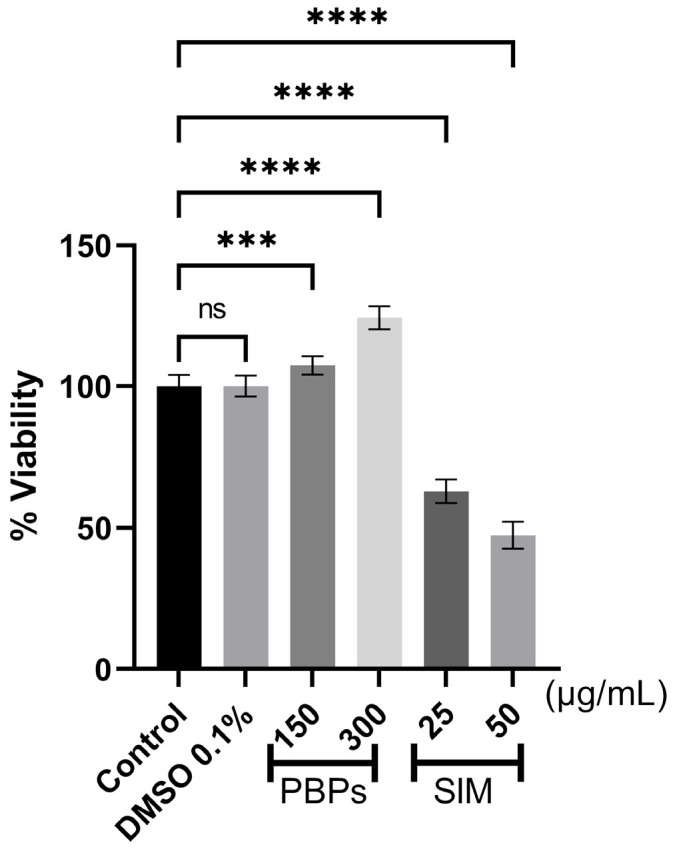
Effect of phycobiliproteins (PBPs) from *A. maxima* and simvastatin (SIM) on the viability of 3T3-L1 preadipocytes at 24 h. Values are shown as mean ± standard deviation (n = 12). Data were compared using a one-way ANOVA analysis with Tukey’s multiple comparison test. Asterisks indicate significantly different means (*** *p* < 0.001 and **** *p* < 0.0001).

Cells treated with PBPs (150 and 300 µg/mL) did not show any detrimental effects on cell viability. On the contrary, they exhibited a significant increase (7% and 24%, respectively) (*p* < 0.001) in cell proliferation compared to untreated preadipocytes (control). Cells treated with simvastatin (25 and 50 µg/mL) showed a significant decrease in cell proliferation compared to the control, by 37% and 52% (*p* < 0.0001), respectively. The results of cells treated with 0.1% DMSO showed no changes in viability compared to untreated cells, suggesting that the observed inhibition of viability can be attributed solely to the effect of simvastatin (Figure 1).

### 2.2. Effect of Treatment with A. maxima Phycobiliproteins (PBPs) and Rosiglitazone (RSG) on Adipogenesis in 3T3-L1 Cells

**Figure 2:**
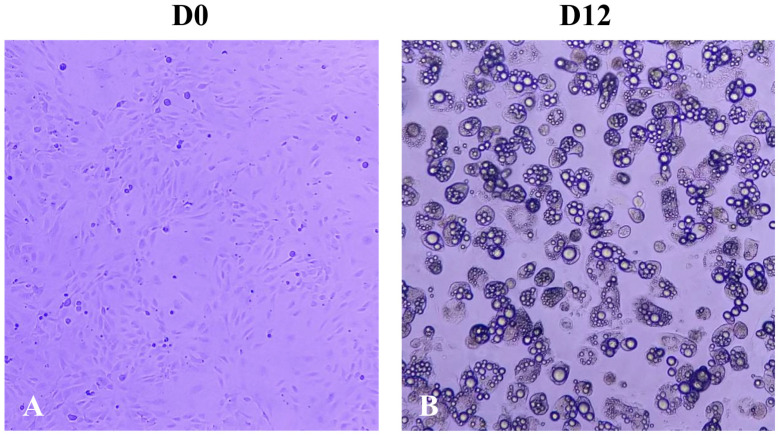
Mouse 3T3-L1 shows the morphology of the non-treated cells on differentiation day (**A**) day 0 (D0) and (**B**) day 12 (D12). Image photographs were taken at ×10 magnification in optical microscopy.

In Figure 2, the morphology of 3T3-L1 cells is observed at day 0 of differentiation (D0) and day 12 (D12). At D0, the typical fibroblast-like morphology characteristic of these preadipocytes can be appreciated. In contrast, at D12, rounded cells with lipid droplets in their interiors are observed, indicating the morphological changes that occur after 12 days of induction of the adipogenic process.

**Figure 3:**
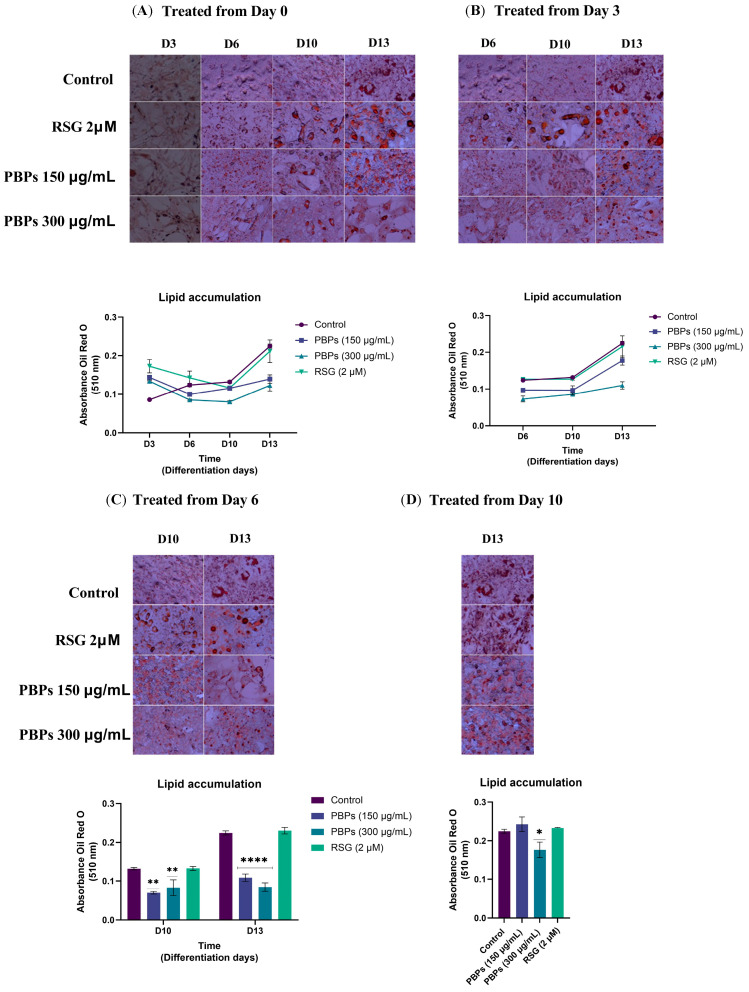
Effect of PBPs on 3T3-L1 adipocyte differentiation. 3T3-L1 fibroblasts were treated with PBPs and RSG from D0 (**A**), D3 (**B**), D6 (**C**), and D10 (**D**), and they were induced to differentiation until D13. Morphological changes and lipid accumulation were documented every three days. Control—cells without treatment; PBPs—phycobiliproteins; RSG—rosiglitazone. Polarized light microscopy images of ORO 0.2% staining—10× magnification; 1× zoom; 100 µm scale. Asterisks indicate significantly different means (* *p* < 0.05; ** *p* < 0.01; and **** *p* < 0.0001).

Figure 3A shows mouse preadipocytes differentiated in culture in the presence of different concentrations of *A. maxima* PBPs and rosiglitazone for 13 days. The untreated control cells displayed a fibroblast-like appearance at D3. However, as adipogenesis progressed, the adipocytes gradually acquired a rounded shape and lipid droplets became visible under the microscope (D6, D10, D13). On day three of differentiation, the cells treated with the three interventions still exhibited a typical fibroblast morphology without the noticeable presence of lipid droplets under the microscope. However, lipid droplets were observed in the cells treated with the interventions by the sixth day compared to the control. At D10, the cells treated with the interventions displayed much larger droplets, and in some cells, a single larger droplet was observed. At D13, all cells presented larger droplets and some cells appeared more rounded (Figure 3A–D).

### 2.3. Effect of A. maxima Phycobiliproteins (PBPs) and Rosiglitazone (RSG) on Adipogenesis in 3T3-L1 Cells after Treatment at Different Time Points during 12 Days of Differentiation

The treatment was initiated on day zero. When the amount of lipid droplets was measured on D3, the treatment with 150 μg/mL of PBPs showed a 67% increase (*p* ≤ 0.01) in lipid droplets compared to the untreated control. Similarly, the treatment with 300 μg/mL of PBPs exhibited a 55% increase (*p* ≤ 0.05) in droplets, while the treatment with RSG (2 μM) displayed a 100% increase (*p* ≤ 0.001) in lipids compared to the untreated control. However, in the following days (D6, D10, and D13), the PBPs treatments showed a lower accumulation of lipids compared to the control. The treatment with 300 μg/mL of PBPs showed significant differences on D6 (31% fewer droplets; *p* ≤ 0.05) and D10 (39% fewer; *p* ≤ 0.01) compared to the control. On D13, both concentrations of PBPs treatment exhibited 38% and 45% fewer lipids than the control (*p* ≤ 0.001). The RSG treatment did not show significant differences on D6, D10, and D13 compared to the control (Figure 3A).

The treatment was initiated on day 3. When the treatments were applied starting from day 3 of the differentiation process, lipid accumulation was measured on days 6, 10, and 13. The treatment with 150 μg/mL of PBPs showed a decreasing trend; however, with 300 μg/mL, there was a significant decrease of 41% in the number of stained lipid droplets compared to the untreated control on D6 (*p* ≤ 0.01). On D10, this treatment exhibited a 35% decrease (*p* ≤ 0.05). During these days, the treatment with 150 μg/mL of PBPs and the RSG treatment did not show significant differences compared to the control. However, 150 μg/mL of PBPs showed 22% and 27% less lipid content compared to the control on D6 and D10, respectively. On D13, 150 μg/mL of PBPs displayed a 22% reduction in lipid droplets compared to the control (*p* ≤ 0.05), and 300 μg/mL of PBPs exhibited a 51% reduction (*p* ≤ 0.001). The RSG treatment did not show significant differences from the control on days 6, 10, and 13 (Figure 3B).

When the treatments with 150 and 300 µg/mL of PBPs were applied starting from day 6 of the differentiation process, there was a lower accumulation of lipids compared to the untreated control on D10 (47% and 37%, respectively) and on D13 (52% and 62%, respectively) (Figure 3C). When the treatments were applied starting from day 9 and lipid accumulation was measured on D13, only the treatment with 300 μg/mL of PBPs showed a 21% decrease in lipid accumulation compared to the control (*p* ≤ 0.05) (Figure 3D). Rosiglitazone did not show any effect during these days.

### 2.4. Effect of A. maxima Phycobiliproteins (PBPs) on the Relative mRNA Expression of Genes Involved in Lipid Metabolism, Glucose Metabolism, Thermogenesis, and Antioxidant Defence in the Adipogenic Process in 3T3-L1 Cells

This study evaluated the effect of three treatments, PBPs (150 µg/mL), RSG (2 µM), and E (10 µM), on the expression of eight genes of interest. Additionally, the treatments were added at different time points during adipogenesis, specifically, day 0, day 3, day 6, and day 9. The expression of the different markers of interest was measured at different time intervals, including 24 h, day 6 (D6), day 9 (D9), and day 12 (D12).

#### 2.4.1. Treatment Initiation from Day 0 of Differentiation

**Figure 4:**
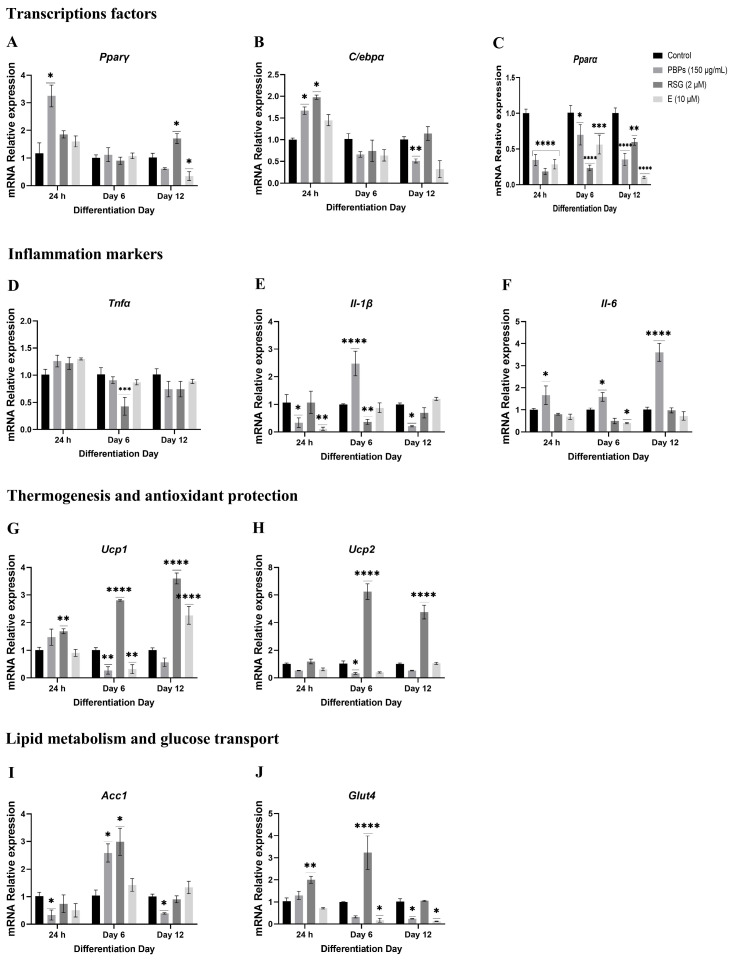
Effect of *A. maxima* phycobiliproteins (PBPs) on gene expression (**A**–**J**) during the adipogenic process in 3T3-L1 fibroblasts when treatments were added on day 0 and collected at 24 h, day 6, and day 12 of the adipogenic process. Untreated cells (Control) and cells treated with rosiglitazone (RSG) and estradiol (E) were used as controls. The constitutive gene *Actin* was used as an internal control for qPCR expression studies. The values are shown as the average ± SEM, (n = 6 per group). The samples were run in triplicate. Data were compared with a two-way ANOVA analysis with the Fisher test for multiple comparisons. * *p* < 0.05; ** *p* < 0.01; *** *p* < 0.001 and **** *p* < 0.0001.

When preadipocytes were treated with PBPs, RSG, and E from day 0 of differentiation, the expression of the genes of interest was measured at 24 h, day 6, and day 12 (Figure 4).

##### mRNA Expression of Markers at 24 h of the Adipogenic Process

It was observed that the treatment with PBPs upregulated *Pparγ* mRNA 1.2-fold (*p* < 0.05). The treatments with RSG and E did not show significant differences compared to the control in the expression of this mRNA, although they showed a tendency towards overexpression (Figure 4A). In the case of *C/ebpα* mRNA, both PBPs and RSG treatments significantly (*p* < 0.05) overexpressed it 0.67-fold and 0.97-fold compared to the control, respectively, while the E treatment had no effect (Figure 4B). On the other hand, all three treatments showed significant (*p* < 0.0001) 0.66-fold, 0.82-fold, and 0.72-fold reductions in the expression of the *Pparα* gene, respectively, compared to the control (Figure 4C). As for *Tnfα* mRNA, the treatments did not influence its expression (Figure 4D). However, both PBPs and E treatments negatively regulated the expression of the *Il-1β* gene 0.75-fold and 0.96-fold, respectively, compared to the control (*p* < 0.05 and *p* < 0.01, respectively). The RSG treatment did not show an effect (Figure 4E). PBPs treatment upregulated *Il-6* mRNA 0.66-fold compared to the control (*p* < 0.05), while RSG and E treatments had no effect (Figure 4F). In the case of *Ucp1* mRNA, the RSG treatment upregulated its expression 0.66-fold (*p* < 0.01), while the PBPs and E treatments had no effect (Figure 4G). On the other hand, the treatments had no effect on the expression of *Ucp2* mRNA (Figure 4H). Finally, in the *Acc1* gene, it was observed that cells treated with PBPs showed significant (*p* < 0.05) 0.7-fold lower expression of this gene compared to untreated cells. The RSG and E treatments did not show significant differences compared to the control (Figure 4I). Additionally, the RSG treatment significantly (*p* < 0.01) overexpressed the *Glut4* gene 0.90-fold compared to the control, while the PBPs and E treatments did not show significant differences compared to the control (Figure 4J).

##### mRNA Expression of Markers at D6 of the Adipogenic Process

Regarding the expression of *Pparγ* and *C/ebpα* mRNA, no significant differences were observed between the treatments and the control (Figure 4A,B). In the case of *Pparα* mRNA, all three treatments (PBPs, RSG, and E) showed downregulation in the expression of this mRNA compared to the control (*p* < 0.05 and *p* < 0.0001). The reduction in mRNA expression was 0.32-fold, 0.77-fold, and 0.45-fold for the PBPs, RSG, and E treatments, respectively (Figure 4C). Regarding *Tnfα* mRNA, the RSG treatment decreased the expression of this mRNA 0.60-fold compared to the control (*p* < 0.0001). Conversely, the PBPs and E treatments did not show significant differences compared to the control (Figure 4D). For *Il-1β* mRNA, the PBPs treatment upregulated the expression of this gene 1.48-fold (*p* < 0.0001), while RSG showed 0.64-fold downregulation compared to the control (*p* < 0.01) (Figure 4E). The E treatment had no effect. In the case of *Il-6* mRNA, the PBPs treatment upregulated the expression 0.56-fold compared to the control (*p* < 0.05), while the E treatment showed a 0.61-fold downregulation (*p* < 0.05). The RSG treatment had no effect (Figure 4F). Regarding *Ucp1* mRNA, both the PBPs and E treatments downregulated the expression of this gene 0.74-fold and 0.7-fold (*p* < 0.01), respectively. In contrast, the RSG treatment overexpressed it 1.78-fold compared to the control (*p* < 0.0001) (Figure 4G). As for *Ucp2* mRNA, the PBPs treatment downregulated the expression of this gene 0.71-fold (*p* < 0.05). In contrast, the RSG treatment overexpressed this messenger 5.02-fold compared to the control (*p* < 0.0001) (Figure 4H). Regarding the expression of *Acc1* mRNA, the PBPs and RSG treatments overexpressed this gene 1.44-fold and 1.83-fold (*p* < 0.05), respectively, compared to the control. The E treatment had no effect (Figure 4I). On the other hand, the RSG treatment overexpressed *Glut4* mRNA 2.23-fold (*p* < 0.0001) compared to the control, while E negatively regulated it 0.83-fold (*p* < 0.05). The PBPs treatments did not show an effect on the expression (Figure 4J).

##### Expression of mRNA of Markers at Day 12 of the Adipogenic Process

The relative expression of the *Pparγ* gene mRNA, compared to the control, was overexpressed by 0.65 times (*p* < 0.05) with RSG treatment. Treatment with E downregulated the expression by 0.68 times (*p* < 0.05). Treatment with the PBPs showed no effect (Figure 4A). The relative expression of *C/ebpα* mRNA was significantly decreased by 0.5 times (*p* < 0.01) with PBPs treatment compared to the control, while RSG and E treatments had no effect (Figure 4B). The relative expression of *Pparα* mRNA was negatively regulated under all treatments, with PBPs, RSG, and E reducing it by 0.65, 0.41, and 0.90 times, respectively (*p* < 0.01 and *p* < 0.0001) (Figure 4C). As for the mRNA expression of *Tnfα*, the treatments had no effect (Figure 4D). In the case of *Il-1β* expression, PBPs treatment downregulated messenger expression by 0.8 times (*p* < 0.05), while RSG and E treatments had no effects (Figure 4E). PBPs treatment overexpressed *Il-6* by 2.55 times (*p* < 0.0001). RSG and E had no effect (Figure 4F). PBPs treatment did not show significant differences in *Ucp1* mRNA expression. RSG and E treatments overexpressed this gene by 2.6 and 1.24 times, respectively, compared to the control (*p* < 0.001) (Figure 4G). PBPs and E treatments had no effect on *Ucp2* expression. RSG treatment overexpressed this messenger by 3.74 times (*p* < 0.0001) (Figure 4H). The expression of *Acc1* mRNA in cells treated with the PBPs significantly decreased by 0.62 times (*p* < 0.05) (Figure 4I). RSG and E treatments had no effect. For *Glut4* mRNA, PBPs and E treatments significantly decreased the expression by 0.79 and 0.9 times (*p* < 0.05), respectively. RSG treatment had no effect (Figure 4J).

#### 2.4.2. Treatment Initiation from Day 3 of Differentiation

**Figure 5:**
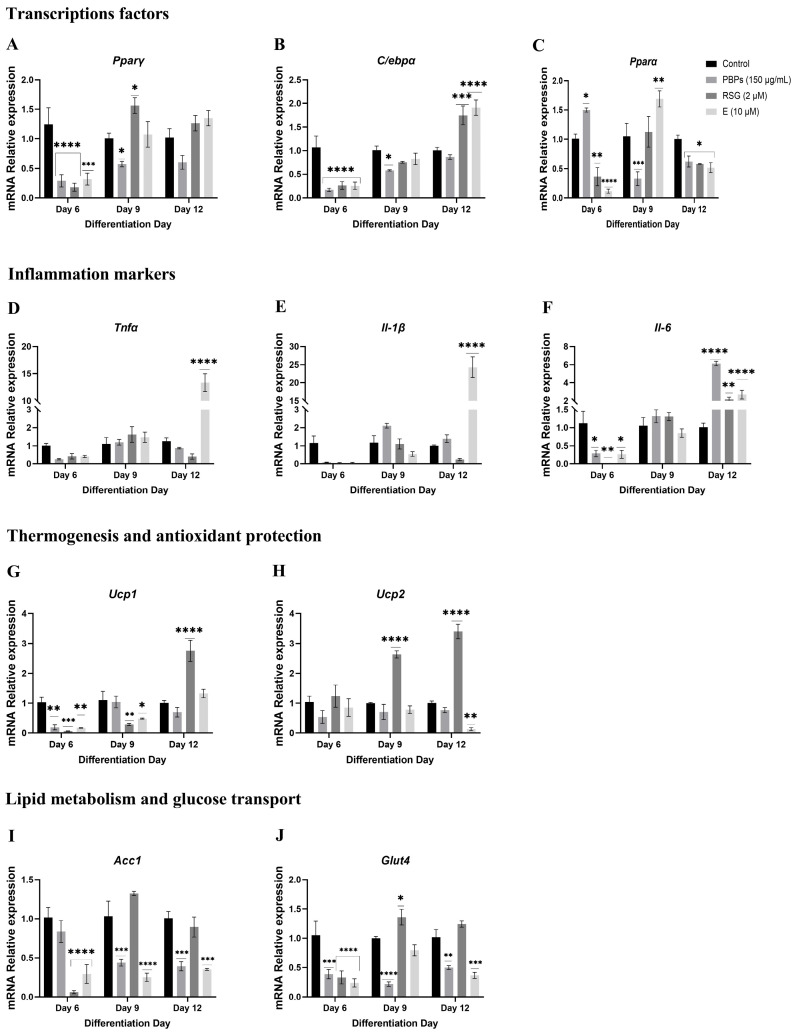
Effect of *A. maxima* phycobiliproteins (PBPs) on gene expression (**A**–**J**) during the adipogenic process in 3T3-L1 fibroblasts, when treatments were added on day 3 and collected on days 6, 9, and 12 of the adipogenic process. Untreated cells (Control) and cells treated with rosiglitazone (RSG) and estradiol (E) were used as controls. The constitutive gene *Actin* was used as an internal control for qPCR expression studies. The values are shown as the average ± SEM (n = 6 per group). The samples were run in triplicate. Data were compared with a two-way ANOVA analysis with the Fisher test for multiple comparisons. * *p* < 0.05; ** *p*< 0.01; *** *p*< 0.001 and **** *p* < 0.0001.

When the preadipocytes were treated with PBPs, RSG, and E from day 3 of differentiation, the expression of different markers was measured at D6, D9, and D12 (Figure 5).

##### mRNA Expression of Markers at Day 6 of the Adipogenic Process

Treatments with PBPs, RSG, and E downregulate the mRNA expression of *Pparγ* by 0.99, 1.1, and 1.0 times, respectively (*p* < 0.0001 and *p* < 0.01), and *C/ebpα* by 0.91, 0.83, and 0.83 times (*p* < 0.0001), respectively, compared to the control (Figure 5A,B). On the other hand, treatment with PBPs overregulated the mRNA expression of *Pparα* by 0.48 times (*p* < 0.05), while RSG and E downregulated its expression by 0.65 and 0.90 times, respectively (*p* < 0.01 and *p* < 0.0001) (Figure 5C). For *Tnfα* and *Il-1β* mRNA, the treatments showed no effects (Figure 5D,E). In contrast, all three treatments (PBPs, RSG, and E) downregulated the expression of the *Il-6* gene by 0.87, 1.11, and 0.89 times, respectively (*p* < 0.05 and *p* < 0.01) (Figure 5F). The same pattern was observed for the expression of *Ucp1* mRNA, with reductions of 0.85, 0.97, and 0.87 times for PBPs, RSG, and E, respectively (*p* < 0.01 and *p* < 0.001) (Figure 5G). The treatments had no effect on the expression of *Ucp2* mRNA (Figure 5H). RSG and E treatments downregulated the expression of the *Acc1* gene by 0.96 and 0.73 times (*p* < 0.0001), respectively, compared to the control. However, treatment with PBPs showed no significant effects on the expression of this gene (Figure 5I). Regarding *Glut4* expression, all three treatments, PBPs, RSG, and E, downregulated it by 0.68, 0.74, and 0.82 times, respectively (*p* < 0.01 and *p* < 0.0001) (Figure 5J).

##### mRNA Expression of Markers at Day 9 of the Adipogenic Process

The mRNA of *Pparγ* was downregulated by PBPs by 0.44 times (*p* < 0.05) compared to the control, while treatment with RSG overregulated it by 0.54 times (*p* < 0.05). On the other hand, treatment with E had no effect (Figure 5A). *C/ebpα* was also negatively regulated by PBPs by 0.43 times (*p* < 0.05) compared to the control. The treatments with RSG and E showed no significant differences compared to the control (Figure 5B). Regarding *Pparα*, treatment with PBPs negatively regulated the expression of this gene by 0.74 times (*p* < 0.001) compared to the control. In contrast, treatment with E positively regulated its expression by 0.55 times (*p* < 0.01). RSG showed no significant differences compared to the control (Figure 5C). There was no effect on the mRNA of *Tnfα*, *Il-1β*, or *Il-6* with any treatment (Figure 5D–F). In the case of *Ucp1*, treatment with RSG and E negatively regulated its expression by 0.84 and 0.66 times (*p* < 0.01 and *p* < 0.05), respectively, while PBPs had no effect (Figure 5G). *Ucp2* was positively regulated by RSG by 1.63 times (*p* < 0.0001) compared to the control, while treatments with PBPs and E had no effect (Figure 5H). Finally, *Acc1* was negatively regulated by treatments with PBPs and E, by 0.61 and 0.79 times its expression compared to the control (*p* < 0.001), respectively. RSG showed no significant differences compared to the control (Figure 5I). In the case of *Glut4*, treatment with PBPs downregulated its expression by 0.78 times compared to the control (*p* < 0.001), while treatment with RSG overregulated it by 0.36 times (*p* < 0.05). Treatment with E showed no significant differences compared to the control (Figure 5J).

##### mRNA Expression of Markers at Day 12 of the Adipogenic Process

The relative mRNA expression of the *Pparγ* gene compared to the control, when treated with PBPs (150 µg/mL) from day 3 and measured at day 12 of adipocyte differentiation, showed a tendency to decrease by 0.43 times compared to the control. Treatment with RSG and E showed no significant differences compared to the control (Figure 5A). The relative mRNA expression of *C/ebpα* after treatment with PBPs was unaffected. Treatments with RSG and E significantly overexpressed this gene by 0.74 and 0.90 times (*p* < 0.001), respectively (Figure 5B). The relative mRNA expression of *Pparα* was reduced when cells were treated with PBPs by 0.38 times (*p* < 0.05), with RSG by 0.42 times (*p* < 0.05), and with E by 0.49 times compared to the control (*p* < 0.05) (Figure 5C). The mRNA expression of *Tnfα* showed no significant differences with PBPs or RSG treatment, while treatment with E overregulated it by 9.36 times compared to the control (*p* < 0.0001) (Figure 5D). PBPs treatments did not have a significant effect on the mRNA expression of *Il-1β*, but there was a tendency to overexpress this gene. On the other hand, RSG tended to decrease the expression of this messenger by 0.76 times compared to the control. Treatment with E overexpressed it by 23.2 times compared to the control (*p* < 0.0001) (Figure 5E). The expression of *Il-6* showed an overexpression with all three treatments (PBPs, RSG, and E) by 5.0, 1.14, and 1.65 times more than the control (*p* < 0.01 and *p* < 0.0001) (Figure 5F). Treatments with PBPs and E had no effect on the mRNA expression of *Ucp1*. Treatment with RSG overexpressed this gene by 1.73 times compared to the control (*p* < 0.0001) (Figure 5G). Treatment with PBPs had no effect on the mRNA expression of *Ucp2*. Treatment with RSG overexpressed this gene by 2.38 times compared to the control (*p* < 0.0001). Treatment with E downregulated it by 0.88 times compared to the control (*p* < 0.01) (Figure 5H). At this point, the mRNA expression of *Acc1* in cells treated with PBPs and E significantly decreased by 0.62 and 0.66 times (*p* < 0.001), respectively, compared to the control. Regarding treatment with RSG, it showed no significant effect (Figure 5I). For the mRNA expression of *Glut4*, treatments with PBPs and E significantly decreased the expression by 0.53 and 0.66 times (*p* < 0.01 and *p* < 0.001), respectively. RSG had no significant effect compared to the control (Figure 5J).

#### 2.4.3. Treatment Initiation from Day 6 (T_D6) and Day 9 (T_D9) of Differentiation

**Figure 6:**
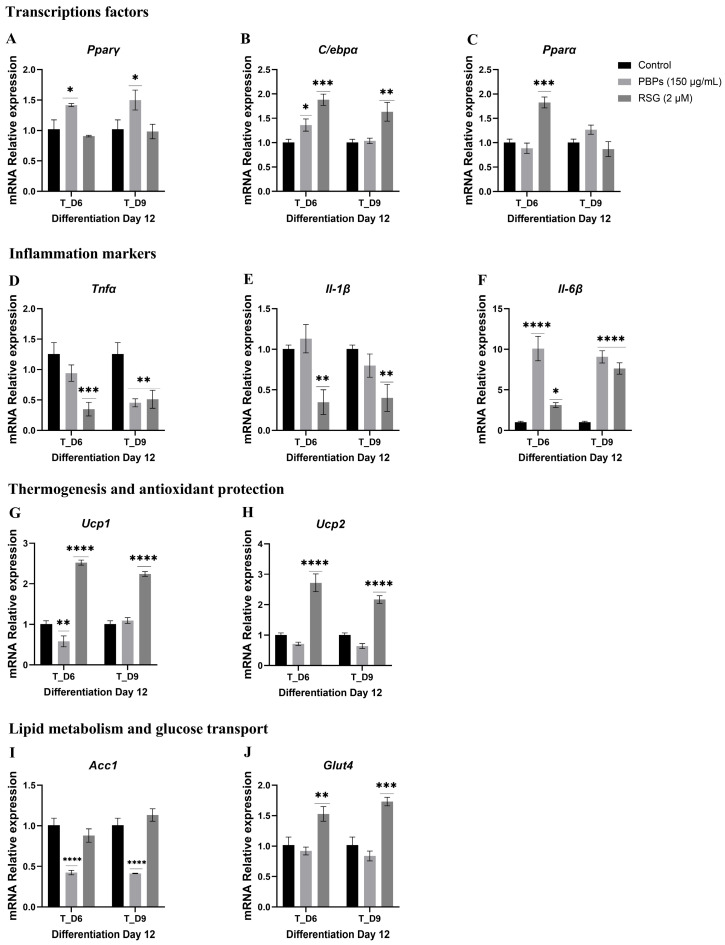
Effect of *A. maxima* phycobiliproteins (PBPs) on gene expression (**A**–**J**) during the adipogenic process in 3T3-L1 fibroblasts, when treatments were added on days 6 and 9, and collected on day 12 of the adipogenic process. Untreated cells (Control) and cells treated with rosiglitazone (RSG) and estradiol (E) were used as controls. The constitutive gene *Actin* was used as an internal control for qPCR expression studies. The values are shown as the average ± SEM (n = 6 per group). The samples were run in triplicate. Data were compared with a two-way ANOVA analysis with the Fisher test for multiple comparisons. * *p* < 0.05; ** *p* < 0.01; *** *p* < 0.001 and **** *p* < 0.0001.

When preadipocytes were treated from T_D6 and T_D9 of differentiation, the expression of different markers was measured until day 12 (Figure 6).

**Figure 7:**
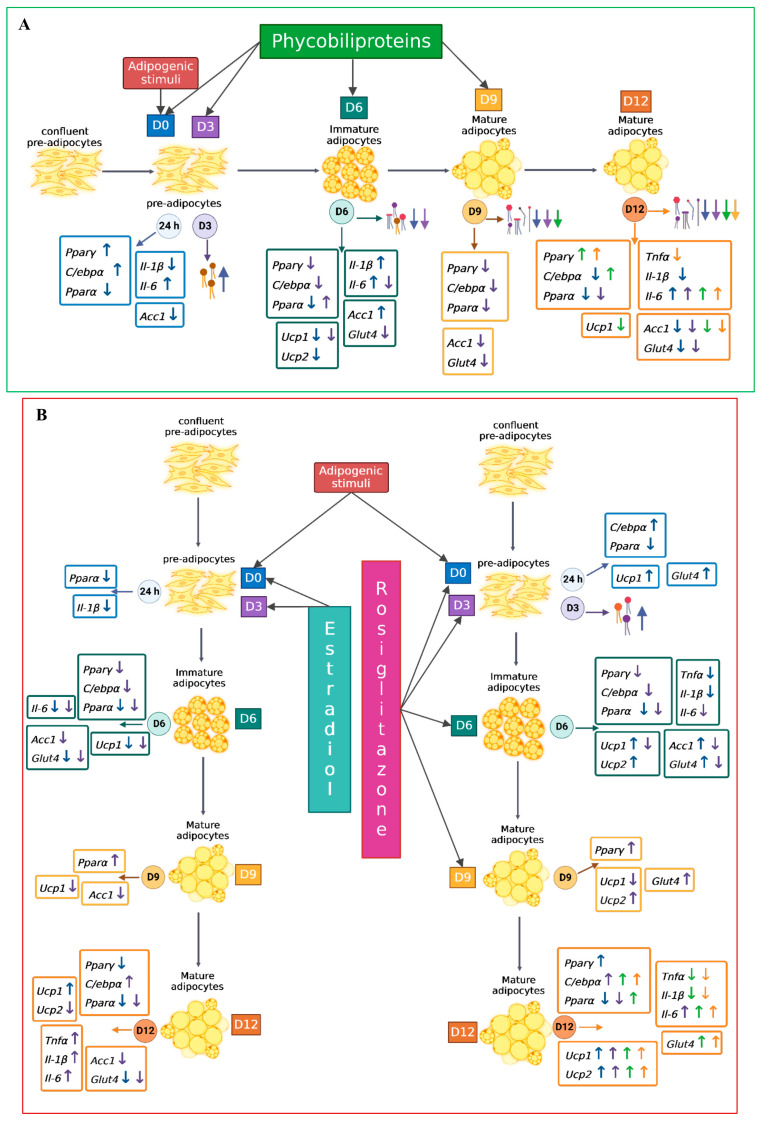
The effects of (**A**). phycobiliproteins (PBPs) of *A. maxima*, (**B**). rosiglitazone (RSG), and estradiol (E) on lipid accumulation and gene expression of adipogenesis and inflammation on the days from which the treatment (D0, D3, D6, and D9) was added, along with the middle, with changes every other day. The colored circles represent the days when lipid droplet accumulation in cells and gene expression were measured (24 h, D3, D6, D9, and D12). In the rectangles of colored lines, the different genes studied are divided according to the categories established in the figures corresponding to gene expression. The colored arrows indicate the upregulation (↑, ↑, ↑, ↑) or downregulation (↓, ↓, ↓, ↓) of treatments in the mRNA of each gene, and their color is also related to the day the treatment was added. Lipids are represented by commonly used symbols for saturated, unsaturated, monoglyceride, diglyceride, and triglyceride fatty acids. The classification of adipocytes was made as reported by Esteve Ràfols, (2014) [21]. Only the results that presented significant differences with respect to their respective controls were placed.

The relative expression of the *Pparγ* mRNA gene compared to the control, when treated with 150 µg/mL of PBPs from days 6 and 9, was significantly upregulated by 0.38 and 0.46 times (*p* < 0.05), respectively, compared to untreated cells. The treatment with RSG had no effect (Figure 6A). The relative expression of the *C/ebpα* mRNA gene with PBPs at T_D6 was 0.36 times higher compared to untreated cells (*p* < 0.05), while it was unaffected at T_D9. Treatment with RSG at T_D6 and T_D9 showed significantly higher expression by 0.87 and 0.63 times (*p* < 0.001 and *p* < 0.01), respectively, compared to the control (Figure 6B). The relative expression of the *Pparα* mRNA gene showed no significant differences from the control when cells were treated with PBPs. Treatment with RSG at day 6 (T_D6) upregulated the gene by 0.81 times (*p* < 0.001), while it had no significant effect at T_D9 compared to the control (Figure 6C). PBPs had no significant effect on the expression of the *Tnfα* mRNA at T_D6, but at T_D9, its expression decreased by 0.89 times compared to the control (*p* < 0.01). RSG at T_D6 and T_D9 decreased the expression of this mRNA by 0.97 and 0.84 times (*p* < 0.001 and *p* < 0.01), respectively, compared to the control (Figure 6D). Regarding the expression of *Il-1β* mRNA, treatment with PBPs at T_D6 and T_D9 had no effect. Treatment with RSG at T_D6 and T_D9 significantly decreased the expression of this mRNA by 0.66 and 0.60 times (*p* < 0.01), respectively, compared to the control (Figure 6E). Treatment with PBPs at T_D6 and T_D9 enhanced the expression of *Il-6* by 8.94 and 7.93 times (*p* < 0.001), respectively, compared to the control. Additionally, RSG at T_D6 and T_D9 upregulated this gene by 2.06 and 6.52 times (*p* < 0.05 and *p* < 0.001), respectively, compared to the control (Figure 6F). Treatment with PBPs significantly reduced the expression of the *Ucp1* mRNA gene by 0.43 times (*p* < 0.01). In contrast, treatment with RSG upregulated this gene by 1.5 times compared to the control (*p* < 0.001). At T_D9, treatment with phycobiliproteins had no effect, but RSG upregulated this mRNA by 1.22 times compared to the control (*p* < 0.001) (Figure 6G). Treatment with phycobiliproteins had no effect on the expression of the *Ucp2* mRNA gene. Treatment with RSG at T_D6 and T_D9 significantly upregulated this gene by 1.7 and 1.2 times (*p* < 0.001), respectively, compared to the control (Figure 6H). The expression of the *Acc1* mRNA gene in cells treated with PBPs at T_D6 and T_D9 significantly decreased by 0.59 and 0.60 times (*p* < 0.001), respectively, compared to the control. Regarding RSG treatment, it had no effect (Figure 6I). For the *Glut4* mRNA gene, treatment at D6 and D9 showed no significant differences compared to the control. RSG treatment at T_D6 and T_D9 significantly upregulated the gene by 0.48 and 0.68 times (*p* < 0.01 and *p* < 0.001), respectively, compared to the untreated control (Figure 6J). All the significant results obtained in this work are summarized in Figure 7A,B.

## 3. Discussion

Marine-active products are safe and effective in the treatment of obesity. Phytochemicals can ameliorate obesity through mechanisms including negative regulation of adipogenesis and positive regulation of thermogenesis [22]. This study evaluated the effect of *A. maxima* phycobiliproteins, rosiglitazone, and 17b-estradiol as regulatory controls, on lipid accumulation in 3T3-L1 cells, as well as the relative mRNA expression of key transcription factors involved in the adipogenic process, markers of inflammation, thermogenesis, antioxidant effect, lipid metabolism and glucose metabolism during distinct phases of adipogenesis. RSG, a thiazolidinedione (TZD) and *Pparγ* agonist used clinically as an antidiabetic agent, was used as a positive control for adipogenesis. Activation of *Pparγ* by TZDs (rosiglitazone, pioglitazone, and troglitazone) enhances WAT expansion, alleviates peripheral lipotoxicity, and normalizes adipokine secretion [23]. In this study, E was used as a negative control for adipogenesis because it interferes with the actions of *Pparγ* on adipogenesis both in vivo and in vitro. This effect is achieved through negative regulation of adipogenesis-related genes, which is mediated by the inhibition of coactivator recruitment by *Pparγ* [9].

The differentiation of adipocytes and the expansion of adipose tissue in response to excessive calorie intake are crucial for preventing the abnormal accumulation of fats in non-adipose tissues. Research conducted on both mice and humans provides evidence that this ectopic lipid deposition contributes to the development of metabolic disorders like hyperlipidemia and insulin resistance. When adipocytes increase in size and/or number excessively, they release higher amounts of molecules such as adipokines, free fatty acids, and inflammatory mediators, which impact various tissues including the liver, muscles, and neural connections, thereby leading to obesity-related complications [24].

In the present work, the results show that PBPs induce an increase in the number of pre-adipocytes 24 h after treatment. Induction of cell proliferation has been shown to have an impact on the storage capacity of adipose tissue by generating new adipocytes. In addition, increasing the number of healthy adipocytes reduces insulin resistance in adipose tissue. Distributing the same amount of fat among a greater number of cells leads to a decrease in *Tnfα* levels and insulin resistance. This principle has been one of the cornerstones of the treatment of T2DM with TZDs, which act on the nuclear receptor *Pparγ* to promote the differentiation of new adipocytes [25].

Applying a differentiation stimulation regimen consisting of cAMP, insulin, and glucocorticoids allows these cells to differentiate within a period of 4 to 6 days. Approximately 4 days after adding the differentiation-inducing agents, the cells begin to accumulate lipids in the form of lipid droplets, which increase in number and size over time [26]. In this study, it was observed that on day 3 of differentiation, cells treated with all three substances still exhibited a fibroblast morphology. Lipid accumulation measurement with Oil Red O staining showed that on day 3 of differentiation, adding PBPs and RSG from day 0 resulted in greater lipid droplet accumulation compared to the control. However, at all other treatment time points (days 3, 6, and 10), PBPs reduced lipid levels in the cells, and cells treated with RSG did not show any differences compared to the control. A similar result was shown in a previous study using a 70% ethanol extract of *A. maxima* (SM70EE), which contained 59.94% proteins and dose-dependently inhibited lipid accumulation in 3T3-L1 cells after eight days of differentiation. Additionally, phycocyanin reduced the accumulation of lipid droplets in 3T3-L1 adipocytes compared to differentiated control adipocytes [20]. It has also been reported that adding RSG to the medium on the first day of differentiation increases lipid accumulation [27]; conversely, adding it to mature adipocytes reduces lipid accumulation in the cells [28]. In this study, when PBPs were added to preadipocytes, their effect was similar to that of RSG in these cells.

The process of differentiation of preadipocytes into adipocytes is coordinated by a complex transcriptional cascade involving several regulatory factors. The nuclear receptor γ, activated by peroxisome proliferator-activated receptor (*Pparγ*), and several members of the C/EBP family of transcription factors play key roles in this cascade and contribute to regulating the differentiation process of adipose cells. These regulatory factors coordinate the different stages of differentiation, allowing cells to develop into mature adipocytes capable of storing fat and contributing to the increase in adipose tissue in the body [29]. It has been reported that in 3T3-L1 cells, *Pparγ* reaches its highest expression peak on the eighth day of differentiation, while *C/ebpα* peaks on the fourth day [24]. On the other hand, in human SGBS cells, isolated from the adipose tissue of a patient with Simpson–Golabi–Behmel syndrome (SGBS) and widely used in adipocyte differentiation studies, a later peak of expression has been observed for these genes, with day 12 for *Pparγ* and day eight for *C/ebpα* [30]. Our results showed a peak of expression for both genes at day 12 of differentiation. It was observed that when preadipocytes were exposed to PBPs, they overexpressed *Pparγ* within 24 h of being added to the cells along with the differentiation medium on day zero of the process. At the same time point, both PBPs and RSG significantly overexpressed *C/ebpα* mRNA. These results could explain why, on the third day of differentiation, cells treated with PBPs and RSG exhibited greater lipid accumulation compared to the control. Thus, PBPs from *A. maxima* may contribute to the turnover of hypertrophic or necrotic adipocytes into new healthy adipocytes by activating *Pparγ* and *C/ebpα* in preadipocytes. It is worth noting that this activation by PBPs was only observed at 24 h, specifically in preadipocytes, but not in immature adipocytes (day 6) or mature adipocytes (day 12) when the treatment was applied from day zero. Similarly, it has been reported that a protein extract and phycocyanin from *Arthrospira* decreased the expression of C/EBPα and PPARγ proteins in 3T3-L1 cells when added from day zero of differentiation and cultured for eight days [20].

When the treatment with PBPs was added on the third day of differentiation, while the cells were still preadipocytes, it was observed that PBPs negatively regulated the expression of both *Pparγ* and *C/ebpα* mRNA in immature adipocytes (day 6) and mature adipocytes (days 9 and 12). These findings align with the results obtained from treatments added on day 0. When the treatments were added to immature adipocytes (day 6), PBPs upregulated the expression of both *Pparγ* and *C/ebpα*, while RSG only upregulated *C/ebpα*. However, when the culture predominantly consisted of mature adipocytes (day 9), PBPs only overexpressed *Pparγ* mRNA, while RSG exclusively upregulated *C/ebpα*. Based on these observations, we can suggest that the effects of PBPs vary depending on the cell’s stage, either by contributing to cellular turnover through the activation of *Pparγ* and *C/ebpα* or by reducing lipid droplet accumulation through the downregulation of adipogenic and lipogenic markers in 3T3-L1 cells. Furthermore, it was observed that RSG overexpressed *Pparγ* mRNA until day 12 of differentiation when the treatment was added from day 0, indicating that it influenced early overexpression of *C/ebpα* mRNA followed by *Pparγ*.

When RSG was added from day 3, a significant overexpression of *Pparγ* mRNA was observed on day 9, and a trend towards upregulation was seen on day 12. Additionally, both RSG and estradiol showed significant overexpression of *C/ebpα* mRNA on day 12. It has been reported that treatment with RSG (1 μM) in mature adipocytes, added to the cells from the beginning of the adipogenic process (day 0) for 72 h at the initiation of adipocyte differentiation, followed by a regular differentiation protocol until day 14, significantly increased the expression of both *Pparγ* and *C/ebpα* mRNA. However, when adipocytes were cultured until day 14 and treated for 6 days with fresh medium containing RSG, the expression of both genes was reduced compared to the control [27]. On the other hand, treatment with E from the beginning of the differentiation process has a negative effect on the expression of *Pparγ* and *C/ebpα* mRNA, especially on day 12. It has been reported that E treatment inhibited lipid accumulation and the expression of specific adipocyte genes caused by TZDs, such as troglitazone, in 3T3-L1 cells. E interfered with *Pparγ* actions on adipogenesis by negatively regulating adipogenesis-related genes, which are mediated through the inhibition of troglitazone coactivator recruitment for *Pparγ* [9].

*C/ebpα*, like *Pparγ*, is a master transcription factor of adipogenesis. *Pparγ* can induce adipogenesis in cells deficient in *C/ebpα*, while *C/ebpα* is unable to drive the adipogenic programme in the absence of *Pparγ*. This observation suggests that *C/ebpα* and *Pparγ* participate in a single pathway of adipose development, where *Pparγ* is the dominant factor. However, *C/ebpα* plays a critical role during terminal adipogenesis, as the lack of *C/ebpα* expression leads to insulin resistance in cell culture models and an inability to develop WAT in vivo [31]. The PBPs (D6 treatment), along with RSG (D3, D6, and D9 treatment) and E (D3 treatment), were able to positively regulate the expression of *C/ebpα* when measured in mature adipocytes (D12).

The PBPs, RSG, and E negatively regulated the relative expression of *Pparα* mRNA, except for when PBPs and E were added on day 3 and measured on days 6 and 9, respectively, which positively regulated the mRNA of this gene. *Pparα* is predominantly expressed in the liver and, to a lesser extent, in muscles, the heart, bones, and brown adipose tissue (BAT), all of which are highly oxidative tissues rich in mitochondria. It is known that *Pparα* plays an important role in the β-oxidation of fatty acids in these tissues [22]. However, its role and function in WAT are not well understood.

Hinds et al. (2022) [32] reported a specific knockout of *Pparα* (PparaFatKO) in adipose tissue in mice to determine the signaling position of *Pparα* in the expansion of adipose tissue during the development of obesity. In female mice, no changes in adiposity were observed compared to control mice, perhaps due to the protective action of estrogens on adipocyte hypertrophy. However, in male mice, the lack of *Pparα* signaling in adipocytes caused a significant increase in cholesterol esters, activation of the transcription factor SREBP-1, and a shift in macrophage polarity from an anti-inflammatory type to a pro-inflammatory type. This led to increased production of proteins involved in fatty acid synthesis and cholesterol metabolism, as well as an increase in inflammation markers in macrophages. These results indicate that *Pparα* plays an important role in protecting against fat accumulation, inflammation, and imbalances in cholesterol metabolism in adipose tissue.

ACC1 plays an important role in fatty acid biosynthesis, glucagon secretion, lipogenesis, and glucose metabolism. Combating obesity can be achieved not only by reducing lipid consumption but also by restricting adipogenesis. ACC and FAS are important enzymes involved in catalyzing fatty acid synthesis [22]. Furthermore, PBPs overexpressed the mRNA of this gene on day 6 when the treatment was added on day 3 of differentiation. In contrast, RSG positively regulated the mRNA of *Acc1* when the treatment was added on day 6 and measured on day 12, and E showed a similar effect when the treatment was added on day 3 and measured on day 9. However, none of these treatments showed a direct effect on increasing lipid accumulation in the cells.

However, changes in the expression of *Pparα*/γ, *C/ebpα*, and even *Acc1* mRNAs at different time points may be attributed to lipid remodeling occurring during the differentiation of these cells. Miehle et al. (2020) [30] found variation in lipid classes during the adipogenic process in human SGBS cells. This variation depends on the specific requirements of the cells. For instance, these authors reported an increase in ceramide concentrations during the differentiation process, which is known to be involved in cell differentiation signaling. However, these ceramide species decreased after completing differentiation around Day 4, while massive lipid remodeling occurred during adipocyte maturation. This maturation phase was characterized by substantial synthesis of diacylglycerols and triacylglycerols. Additionally, they observed increases in membrane lipids such as phosphatidylcholines, phosphatidylethanolamines, and sphingomyelins, as well as their biosynthetic precursors. Furthermore, studies like that of Halama et al. (2016) [24], which assessed the transcriptome during adipogenesis, provide insights into the genes expressed and the changes occurring in the cells at each stage of differentiation. For example, they noted that genes involved in adipocyte differentiation, adipose tissue size and mass, lipid synthesis and concentration, metabolism, carbohydrate transport and absorption, insulin resistance, and obesity show an ascending pattern of expression from day 2 to day 18, with the least expression on day 2 and the highest expression on day 18. In contrast, genes involved in cell cycle, cell growth and proliferation, and cell differentiation are overexpressed in a descending pattern from day 2 to day 6, with maximum expression on day 2 and minimum expression on day 6. Furthermore, these genes are no longer expressed from days 6 to 8.

Dysregulation of inflammation contributes to the development of inflammatory and metabolic diseases such as atherosclerosis, non-alcoholic fatty liver disease, and diabetes. Therefore, it is important to identify anti-inflammatory agents for the prevention and/or therapy of inflammatory diseases [33]. In this study, we evaluated the effect of PBPs on the inflammation markers *Tnfα*, *Il-1β*, and *Il-6*. Phycobiliproteins downregulated the expression of *Tnfα* and *Il-1β* mRNAs. Previously, it has been reported that an extract of *A. platensis* suppressed the expression and secretion of pro-inflammatory cytokines in macrophages and splenocytes by inhibiting the NF-κB pathway [34]. These results demonstrate the anti-inflammatory properties of PBPs. Interestingly, it was also observed that, at all-time points, PBPs upregulated the mRNA expression of *Il-6*. The homeostatic role versus the pathogenic role of *IL-6* in various autoimmune diseases has been widely debated [35]. *IL-6* is a cytokine with pleiotropic effects due to the ubiquitous presence of *IL-6* receptors, whether cellular or soluble, making most cells potential target cells. Therefore, it is not surprising that many different effects of *IL-6* have been reported. It is a pro-inflammatory cytokine in most cells but can also be anti-inflammatory in some cells and antagonize the effect of TNF-alpha [36]. *IL-6* has been identified as a key regulator of glucose homeostasis through its effects on the production and secretion of GLP-1 from L and alpha cells and subsequent improvements in insulin secretion. Acute effects are caused by an *IL-6*-mediated increase in GLP-1 exocytosis in L cells, while chronic effects are caused by an *IL-6*-mediated increase in glucose responsiveness and GLP-1 production in L and alpha cells [37]. This function of *IL-6* could connect the effect of phycobiliproteins with a mechanism of glucose homeostasis regulation through this pathway, rather than the regulation of *Glut4*, as according to our results PBPs had no effect or negatively regulated *Glut4* mRNA expression in most of the evaluated time points. On the other hand, estradiol upregulated the mRNA expression of *Tnfα*, *Il-1β*, and *Il-6* when cells were treated on day 3 of differentiation and observed at D12. There is a report in which E caused a dose-dependent increase in the release of *IL-8* from adipose cells. Higher levels of *IL-8* were observed when preadipocytes were treated with increasing concentrations of estradiol. Furthermore, an increase in the release of *IL-8* was observed when adipocytes were treated with a combination of rilpivirine and E, suggesting a significant contribution of E in triggering *IL-8* release [38]. It has also been reported that E induced the release of *IL-6* on day 9 of differentiation but not for *Tnfα* [39].

UCP1 is a proton transporter that primarily plays a role in maintaining body temperature in a cold environment by allowing the mitochondrial membrane potential to translate into heat [40]. Additionally, it is known that white adipose tissue is capable of transdifferentiating into beige adipose tissue, characterized by shared features between brown and white adipocytes, including higher expression of UCP1 [41]. For this reason, the induction of this transdifferentiation represents a potential therapeutic strategy for the treatment of obesity [42]. In this study, PBPs negatively regulated the mRNA expression of *Ucp1* when the treatment was applied on D0 and D6, and the expression was measured on D6 and D12, respectively. For the remaining time points of the process, these cells did not show differences in *Ucp1* mRNA expression compared to the control. A similar result was obtained with E treatment, which negatively regulated the mRNA expression of *Ucp1* on D6 and D9. However, on D12 it showed positive regulation when the treatment was added from D0. RSG overexpressed *Ucp1* mRNA, except when it was added to the cells on D3 and the expression was measured on D9, where negative regulation was observed. The observed overexpression of *Ucp1* in this study, primarily by RSG, reinforces the hypothesis of WAT transdifferentiation into BAT by this drug reported previously, a function that occurs through increased mitochondrial mass and lipid oxidation [7]. On the other hand, PBPs do not appear to induce transdifferentiation in these cells.

On the other hand, UCP2, uncoupling protein 2, is part of the mitochondrial transporter family closely linked to UCP1 [43]. UCP2 is considered an antioxidant because it suppresses the generation of reactive oxygen species (ROS) in mitochondria [39]. Due to this, UCP2 expression is associated with chronic inflammation caused by ROS. These ROS, acting as markers of inflammation, could stimulate lipid production and increase fat accumulation. Therefore, UCP2 plays a key role in the connection between obesity and inflammation, influencing cellular metabolism affected by oxidative stress and inflammatory status [43]. PBPs and E showed downregulation of *Ucp2* on D6 when the treatment was added from D0 in the case of PBPs and on D12 when the treatment was added from D3 for E. Additionally, at other time points in the process, cells treated with these agents did not show differences in *Ucp2* mRNA expression compared to the control. This suggests that these cells did not have an inflammatory state that required *Ucp2* mRNA overexpression. Conversely, RSG treatment positively regulated *Ucp2* mRNA expression throughout the process, except on D1 of treatment, when it showed no effect.

## 4. Materials and Methods

The Arthrospira powder (AEH Spiral Spring, Mexico City, Mexico) and PBPs were obtained using the method described by Guzmán-Gómez et al. (2018) [44], with some modifications. Five grams of *Arthrospira* powder was suspended in 20 mL of phosphate buffer (20 mM, pH 7) in a Nalgene centrifuge tube and stirred at room temperature for 5 min. It was then frozen at −70 °C and subsequently thawed in a water bath for 30 min. It was centrifuged at 18,000 rpm for 30 min at 4 °C using a Beckman Coulter Avanti j-30I centrifuge (Beckman Coulter, Brea, CA, USA). The blue supernatant was collected, and another round of centrifugation was performed at 22,000 rpm. The green precipitate was discarded after each centrifugation step. Finally, the supernatant was collected and stored at 4 °C until lyophilization, and the lyophilized sample was stored at −20 °C. RSG was obtained from Cayman Chemical Co. (Ann Arbor, MI, USA), and simvastatin was obtained from Stress Marq Biosciences Inc. (Victoria, BC, Canada). Both compounds were diluted in 100% DMSO to obtain the final study concentrations, with a final DMSO concentration per well ≤0.1%. The MTT used was the Cayman Chemical Cell Proliferation MTT Kit No. 10009365. Stock solutions of PBPs were prepared in differentiation medium, while ethanol was used for preparing E and RSG. The PBPs were then filtrated prior to use. Working solutions of the treatment drugs and PBPs were prepared by diluting the stock solution.

### 4.1. Cell Culture

The in vitro model used for the experiments was 3T3-L1 preadipocytes (Department of Pharmacology, CUCEI-University of Guadalajara, Guadalajara, Mexico). Mouse 3T3-L1 fibroblasts were grown at 37 °C and 5% CO_2_ in growth medium (GM) consisting of Dulbecco’s modified Eagle’s medium (DMEM) (1×) with high glucose and L-glutamine (Gibco 11965), supplemented with 10% *v*/*v* fetal bovine serum (Gibco 26140-079), 1× nonessential amino acids (Gibco 11140), 1 mM sodium pyruvate (Gibco 11360), and 1% *v*/*v* penicillin-streptomycin (10,000 units/mL; Gibco 30-2300).

### 4.2. Cell Proliferation Assay

To determine the effect of the PBPs (3, 30, 150, and 300 μg/mL) and simvastatin (25 and 50 μg/mL) on the proliferation of 3T3-L1 preadipocytes, the MTT (3-(4,5-dimethylthiazol-2-yl)-2,5-diphenyltetrazolium bromide) assay was used, following the protocol provided by the commercial supplier. Cells were seeded at a density of 5000 cells/well in 96-well plates and incubated for 24 h. Then, the treatments were applied and left for 24 h. Simvastatin was used as a positive control for inhibition, and cells without treatment were used as a negative control. Additionally, a control with 0.1% DMSO was included. After the 24-h incubation, 10 μL of MTT solution (5 mg/mL) was added to each well, and the cells were incubated for 4 h at 37 °C. The formazan crystals were then dissolved using the solvent indicated by the kit (100 μL), and the absorbance was measured at 570 nm. The OD readings were taken using a Synergy H1 BioTeK plate reader (Santa Clara, CA, USA) with Gen5 version 2.06.10 software.

### 4.3. Differentiation of Preadipocytes into Adipocytes

For lipid droplet accumulation measurement, cells were seeded at a density of 10,000 cells/well in 24-well plates. Three days after reaching confluence, cells were exposed to adipogenesis inducers, including 0.5 mM IBMX (3-isobutyl-1-methylxanthine), 0.25 µM dexamethasone, and 0.2 IU insulin in DMEM supplemented with 10% fetal bovine serum and 1% antibiotic–antimycotic mixture, for 72 h (from day 0 to day 3 of differentiation). Subsequently, the medium was replaced with DMEM supplemented with 0.1 IU insulin for 72 h (from day 3 to day 6), and then the medium was changed to growth medium, replacing it every three days until day 13 of differentiation. The study groups were as follows: (a) control group of untreated cells; (b) positive control group of adipogenesis treated with 2 µM of RSG; (c, d) groups of cells treated with 150 µg/mL and 300 µg/mL of PBPs. The cells were treated on day 0 (D0), day 3 (D3), day 6 (D6), and day 10 of differentiation. Fresh treatments were applied every 3 days when the medium was replaced. Adipogenesis was monitored every 3 days until day 13 by staining the cells with Oil Red O (ORO) (0.2%) and observing them under a reflected light microscope Motic, PSM-1000 (Hong Kong, China) with a digital camera Moticam 1080 (Hong Kong, China) and a 10× optical objective and 1× zoom; scale 100 µm. The photo montage was performed using the ImageJ software (ImageJ 1.54g) [45]. Subsequently, ORO was extracted from the cells with 100% isopropanol, and lipid accumulation was measured by absorbance at 570 nm.

For gene expression analysis, the cells were seeded at a density of 40,000 cells per well in 6-well plates. Three days later (80% confluence), the cells were exposed to adipogenesis inducers with IBMX (3-isobutyl-1-methylxanthine) at 0.5 mM, dexamethasone at 0.25 µM, and insulin at 0.2 IU in DMEM supplemented for 72 h (from D0 to D3 of differentiation). Then, the medium was replaced with DMEM supplemented with insulin at 0.1 IU for 72 h (from D3 to D6), and thereafter, the medium was replaced with growth medium (supplemented DMEM) every three days until day 12 of differentiation. The study groups were composed as follows: (a) control group of untreated cells; (b) group of cells treated with 150 µg/mL of PBPs; (c) positive control group for adipogenesis treated with 2 µM of RSG; (d) inhibition control group treated with 10 µM E. The treatments were applied to the cells on days 0 (D0), 3 (D3), 6 (D6), and 9 (D9) of differentiation. Cells were collected on D1 (24 h) for RNA extraction from preadipocytes, on D6 for immature adipocytes, and on D9 and D12 for mature adipocytes.

### 4.4. Real-Time Quantitative Reverse Transcription-PCR (qRT-PCR)

The total RNA extraction from 3T3-L1 preadipocytes and adipocytes was performed using the E.Z.N.A. Total RNA Kit II according to the manufacturer’s protocol (Omega BIO-TEK, Norcross, GA, USA). The quantification (OD-260) and quality (OD-260/OD-280) of the RNA were determined using a NanoDropTM 2000/2000c spectrophotometer (Thermo Scientific, Inc., Madison, WI, USA). The integrity of the RNA was verified by electrophoresis on a 1% agarose gel under denaturing conditions. All RNA samples were stored at −70 °C for further use. The cDNA synthesis was performed according to the protocol of the first strand cDNA synthesis kit (Thermo Scientific K1612) using approximately 300 ng of total RNA and oligo(dT)18 primers for a final reaction volume of 20 µL. The real-time polymerase chain reaction (qPCR) was performed in triplicates using a Rotor-Gene Q instrument (QIAGEN, Hilden, Germany) with Maxima SYBR Green/ROX qPCR Master Mix (2×) (Thermo Scientific K0221). The reaction mixture was prepared using 1 µL of cDNA. Each reaction included a negative control, consisting of a reaction without reverse transcriptase, and a no-template control (NTC) to monitor for contamination. The relative expression levels of the target mRNA genes were normalized to the actin gene as a reference gene. The reaction conditions were as follows: initial incubation at 95 °C for 10 min, followed by 50 cycles of denaturation at 95 °C for 10 s, annealing at 50 °C–60 °C for 30 s, extension at 72 °C for 60 s, and a final cooling step at 37 °C for 30 s. The relative gene expression was determined using the 2^−ΔΔCT^ method [46]. The primer sequences used were as follows: *Pparγ* F 5′-TCGCTGATGCACTGCCTATG-′3, R 5′-GAGAGGTCCACAGAGCTGATT-′3, *C/ebpα* F 5′-TTCGGGTCGCTGGATCTCTA-′3, R 5′-TCAAGGAGAAACCACCACGG-′3, *Pparα* F 5′-TTTAGAAGGCCAGGACGATCT-′3, R 5′-GCACTGGAACTGGATGACAG-′3, *Tnfα* F 5′-TCTTCTCATTCCTGCTTGTGG-′3, R 5′-GGTCTGGGCCATAGAACTGA-′3, *Il-1β* F 5′-CAACCAACAAGTGATATTCTCCATG-′3, R 5′-GATCCACACTCTCCAGCTGCA-′3, *Il-6* F 5′-GAGGATACCACTCCCAACAGACC-′3, R 5′-AAGTGCATCATCGTTGTTCATACA-′3, *Ucp1* F 5′-AGTACCCAAGCGTACCAAGC-′3, R 5′-CACACACAGGCGCCTTAAAC-′3, *Ucp2* F 5′-AGCAGTTCTACACCAAGGGC-′3, R 5′-TGGAAGCGGACCTTTACCAC-′3, *Acc1* F 5′-GATCCCCATGGCAATCTG-′3, R 5′-ACAGAGATGGTGGCTGATGTC-′3 and *Glut4* F 5′-GTAACTTCATTGTCGGCATGG-′3, R 5′-AGCTGAGATCTGGTCAAACG-′3. *Actin* F 5′-GTACCCAGGCATTGCTGACA-′3, R 5′-GTTGCTCTGACAACCACAGG-′3.

### 4.5. Statistical Analyses

#### 4.5.1. Cell Proliferation

The experiments were performed in three independent replicates with four replicates each. The results are expressed as mean values ± standard deviation. Statistical analysis was determined by ANOVA followed by Tukey’s multiple comparison test, considering *p* ≤ 0.05 as significant. The statistical analysis was conducted using GraphPad Prism 9 software.

#### 4.5.2. Lipid Accumulation and Gene Expression

The ORO absorbance and gene expression data were expressed as mean values ± standard error of the mean (SEM) (n = 6). The normality of all data was tested using the Shapiro–Wilk and Levene tests. A one-way ANOVA followed by the Fisher’s test was performed for multiple comparisons of all quantitative variables, with a significance level of *p* < 0.05 for a significant difference. Statistical analysis and figure construction were performed using GraphPad Prism 9 software (GraphPad Software, San Diego, CA, USA).

## 5. Conclusions

Overall, the results showed that phycobiliproteins promote cell proliferation within 24 h of treatment. Additionally, regardless of the timing of treatment, the highest number of markers were regulated on days 6 and 12 of differentiation for all three treatments. Phycobiliproteins reduced lipid droplet accumulation on days 3, 6, 10, and 13 of the adipogenic process, while rosiglitazone showed no significant differences compared to the control. On day 6, both phycobiliproteins and rosiglitazone upregulated *Acc1* mRNA. However, all three treatments downregulated *Pparγ* and *C/ebpα*. Phycobiliproteins and estradiol also downregulated *Ucp1* and *Glut4* mRNAs. On the other hand, rosiglitazone, and estradiol downregulated Ppara and *Il-6* mRNAs. On day 12, phycobiliproteins and rosiglitazone upregulated *Pparγ* mRNA and negatively regulated *Tnfα* and *Il-1β* mRNAs. Additionally, phycobiliproteins and estradiol upregulated *Il-6* mRNA and negatively regulated Ppara, *Ucp2*, *Acc1*, and *Glut4* mRNAs. Meanwhile, rosiglitazone and estradiol positively regulated *C/ebpα* and *Ucp1* mRNAs. The regulation of marker mRNA expression by phycobiliproteins was dependent on the cellular differentiation phase. These results demonstrate that phycobiliproteins have an anti-adipogenic and anti-inflammatory effect by reducing the expression of adipogenic, lipogenic, and inflammatory genes in 3T3-L1 cells at different stages of the differentiation process.

## Data Availability

Experiment databases are available upon request.
